# Intelligent Bus Platoon Lateral and Longitudinal Control Method Based on Finite-Time Sliding Mode

**DOI:** 10.3390/s22093139

**Published:** 2022-04-20

**Authors:** Lingli Yu, Yu Bai, Zongxv Kuang, Chongliang Liu, Hao Jiao

**Affiliations:** 1 School of Automation, Central South University, Changsha 410083, China; qq4982113@163.com (Y.B.); qq3061487269@163.com (Z.K.); 2 Beijing Institute of Automation Equipment, Beijing 100074, China; bridge968@sina.com (C.L.); jiaohao@163.com (H.J.)

**Keywords:** intelligent bus platoon, Frenet coordinate system, finite-time tracking control, finite-time stability, string stability

## Abstract

Considering the rapid convergence of the longitudinal and lateral tracking errors of the platoon, a finite-time tracking control method for the longitudinal and lateral directions of the intelligent bus platoon is proposed. Based on the bus platoon model and desired motion trajectory, a distributed longitudinal and lateral finite-time sliding mode tracking control framework of the platoon is designed. Considering the finite-time convergence of the sliding mode of the system, a nonsingular integral terminal sliding mode (NITSM) is designed. An adaptive power integral reaching law (APIRL) is proposed for the finite-time accessibility of the system approaching mode. Based on NITSM-APIRL, a distributed longitudinal and lateral finite-time sliding mode tracking controller for the bus platoon is designed, and a Lyapunov function is created to analyze the finite-time stability and string stability of the system. Based on the Trucksim/Simulink joint simulation experiment platform, the control performance of the method is contrasted with the existing methods, and the actual vehicle test verification is completed by relying on the National Intelligent Connected Vehicle testing zone, which proves the practicability of the method.

## 1. Introduction

The automobile is an important product of human civilization, and it has greatly changed the way of human travel. However, the advent of the automobile also brought new problems, such as traffic jams, energy waste, and carbon emissions [1]. The intelligent vehicle platoon uses Vehicle-to-Everything (V2X) to connect intelligent driving vehicles, which can simplify the complex traffic environment and solve the strong interaction between environmental vehicles. Research has shown that the platooning of vehicles is conducive to improving the stability of traffic flow, alleviating traffic congestion, and reducing energy consumption [2], so the vehicle platoon has an important application potential for solving current traffic problems.

Compared with the construction of a single intelligent vehicle model, the vehicle platoon model needs to consider the interaction between vehicles. The work in [3], which regarded the platoon as a one-dimensional multi-agent system, built a vehicle platoon model based on a four-element architecture. The four-element model does not consider the lateral and longitudinal movement of the vehicle, and uses the vehicle dynamic model, communication topology, geometric configuration of vehicle spacing, and distributed controller to describe the characteristics of the vehicle platoon. The work in [4] assumed that the platoon is driving along an unbiased straight road, and proposed a variable time headway (VTH) strategy. At the same time, the constant distance (CS) strategy and the constant time headway (CTH) strategy are regarded as the specific cases of VTH, and a universal vehicle spacing strategy and vehicle platoon error model are established. The proposed model and method can improve the stability of traffic flow and ensure the stability of the vehicle and platoon. These models can better describe the interaction between vehicles in the platoon from different perspectives, but they all discuss the construction of a one-dimensional platoon model.

Because the one-dimensional platoon model ignores the lateral motion of the vehicle, but in practical applications, the lateral and longitudinal combination of the vehicle needs to be considered. Many scholars have begun to study the construction of the two-dimensional platoon model. The work in [5] constructed a two-dimensional lateral and longitudinal platoon error model for the following leader vehicle structure, used the longitudinal displacement error, lateral position error and heading error of the relative leader vehicle to describe the platoon error state. Then they designed an adaptive platoon control method and used a simulation to verify that the error convergence and anti-disturbance ability of this method are better than traditional linear feedback control. The work in [6] proposed a long short-term memory (LSTM)-based vehicle platoon car-following model, and designed a unidirectional interconnected LSTM model structure, which can reduce the lateral and longitudinal errors of the model by 40%. In [7], considering the effect of car-following and communication delay, a vehicle platoon tracking error model was proposed. Based on this, a distributed nonlinear platoon controller was designed to guarantee that the tracking errors converge to a steady state. The car-following function can avoid negative inter-vehicle spacing errors and negative speeds. The work in [8] proposed a lateral and longitudinal platoon model based on vehicle-mounted radar and vehicle-to-vehicle (V2V) communication, which does not rely on the real-time, high-precision positioning of the vehicle, and which builds a longitudinal model of the platoon based on the relative position feedback of the radar and the CTH strategy. The following vehicle receives the historical data of the preceding vehicle through V2V to estimate its motion trajectory, and then a lateral tracking model of the platoon is designed. The above model describes the lateral and longitudinal motion characteristics of the vehicle platoon by using the location and velocity of the leader vehicle, but ignores the influence of the reference path, resulting in low control accuracy of the vehicle platoon in the curve driving scene, and the follower vehicle cannot track the vehicle ahead normally when the ahead vehicle is out of control.

In order to realize the intelligentization of steering, driving, and braking systems, vehicle platoon tracking control must not only consider multi-vehicle coordination, but also realize lateral and longitudinal cooperation. The work in [9] considers the application cost and proposes a vehicle platoon lateral and longitudinal tracking control method that only uses radar and workshop communication. It uses the extended forward-looking technology to calculate the position, speed, and direction relative to the preceding vehicle, and designs the lateral and longitudinal stable nonlinear controller. The work in [10] uses graph theory to illustrate the construction of the vehicle platoon, uses the potential field method for platoon trajectory planning, and uses kinematics and PID to design the vehicle platoon lateral and longitudinal tracking controllers, which can realize the platoon composition, switching, lane change, obstacle avoidance, and so on. The work in [11] theoretically analyzed the influence of the leader state on the closed loop dynamics of platoon, proving that the leader information can mask out the effect of others and the closed loop dynamics of platoon are equivalent to the leader–follower topology if all followers can receive the leader information and other information. The work in [12] proposed a lateral and longitudinal control method for a nonlinear vehicle platoon based on the consistency theory, constructed a lateral and longitudinal error model relative to the pilot vehicle, and designed a nonlinear joint controller that satisfies the stability of the platoon. The work in [13] studied the lateral and longitudinal tracking control problem of hybrid vehicle platoons, and selected the key points from the trajectory points of manually driven vehicles to form a key point sequence matrix. Using the point set mapping method in real variable function theory, a trajectory correction method was proposed. And a new type of vehicle platoon lateral and longitudinal controller is designed by introducing key point sequence matrix and communication delay. The above methods are all based on the other vehicles in the platoon as a reference. If the other vehicles in the platoon leave the platoon or there is too much tracking error or even tracking failure, the downstream vehicles cannot track the vehicle ahead in a normal fashion.

The lateral and longitudinal control can ensure the joint convergence of the lateral and longitudinal errors, but it cannot guarantee the rapidity of the error convergence. In practical applications, the vehicle platoon must not only ensure the lateral and longitudinal control accuracy, but also pay attention to the rapidity of the system. The member vehicles should respond to the desired motion trajectory or the state changes of other vehicles in time. Regarding the rapidity of error convergence, finite-time sliding mode control is a commonly used technique [14]. The work in [15] analyzed the limitation of the convergence speed of the single-power arrival law when approaching the sliding mode surface, and by introducing a linear power term, a bi-power approximation law is proposed to ensure that the system has the characteristics of global fast approximation. However, the bi-power approximation law cannot make the system state converge to zero, and can only ensure convergence to the steady-state error bound in a limited time, because the system has a bounded perturbation effect. In [16], a distributed coordinated controller, including both lateral and longitudinal motions, is designed for each vehicle with an online estimation of the unknown parameters and disturbances. The work in [17] proposed an integral terminal sliding mode (ITSM) for the conventional terminal sliding mode surface, which is difficult to adapt to the first-order system [18]; therefore, the finite-time stability of the sliding modes can be guaranteed. The work in [19] proposed the fast integral terminal sliding mode (FITSM) based on ITSM, which improved the convergence characteristics of ITSM when it approached the sliding mode surface, and even further improved the convergence speed of the system state. However, both ITSM and FITSM do not consider the control singularity problem in practical applications.

Therefore, this paper proposes a NITSM-APIRL-based lateral and vertical distributed finite-time sliding mode control strategy for bus platoons, aiming at the problems of intelligent bus platoon lateral and longitudinal control and rapid error convergence. The main contributions are as follows: (1) This paper proposed a lateral and longitudinal error model of a bus platoon based on the road coordinate system, which avoids the influence of lateral error on longitudinal displacement calculation; (2) Considering the fast convergence of the sliding mode, NITSM is proposed to solve this problem. In addition, APIRL is proposed for finite-time reachability under bounded disturbance; (3) Based on NITSM-APIRL, a distributed lateral and longitudinal finite-time sliding mode controller is designed to ensure the lateral and longitudinal finite-time stability and platoon stability of a bus platoon. The Trucksim/Simulink co-simulation and real experiments demonstrate the feasibility of the proposed method.

The rest of this paper is arranged as follows. The construction of an intelligent bus platoon model based on the Frenet coordinate system are introduced in Section 2. Furthermore, in Section 3, a bus platoon finite-time sliding mode control method is proposed and then the stability of this method is proved. In Section 4, the simulation and experimental results show the effectiveness and advantage of the proposed control method. Finally, the conclusion and future work are presented in Section 5.

## 2. Construction of Intelligent Bus Platoon Model Based on Frenet Coordinate System

### 2.1. Combined Lateral and Longitudinal Kinematics Model of the Bus

**Figure 1:**
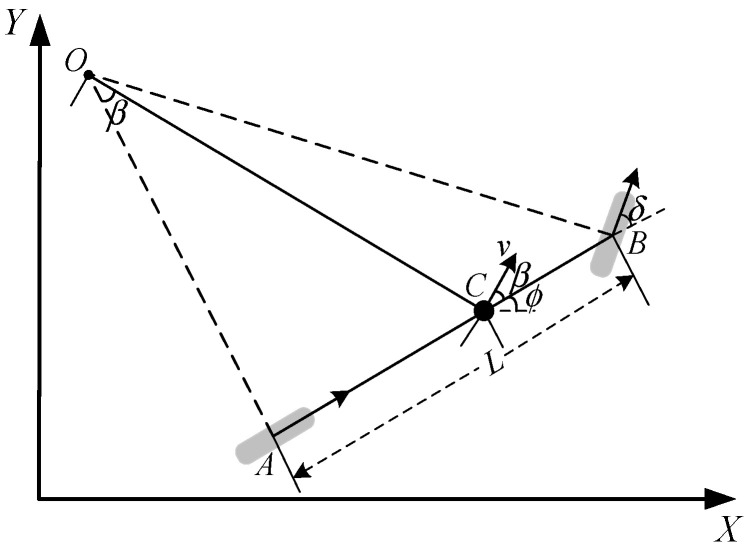
Kinematics Model of Bus Front Wheel Steering.

The front wheel steering kinematics model of the bus is displayed in Figure 1. When the vehicle is driving at a low speed (below 15 m/s) and the turning radius is large (over 20 m), the lateral sliding of the vehicle can be ignored, so the direction of the wheels’ speed can be assumed as the direction of the wheels. At the same time, the vertical lines of the front and rear wheel speed vectors intersect at the steering center O, and the speed vector at the centroid is also perpendicular to the line OC, which connects the steering center to the centroid. Among them, the angle between the centroid velocity vector and the vehicle is the centroid slip angle β, and the angle between the vehicle and the X−axis is the vehicle yaw angle ϕ, L is the vehicle wheelbase, δ is the wheel steering angle, *A* is the center point of the rear wheel of the vehicle, *B* is the center point of the front wheel of the vehicle, and *C* is the center of mass of the vehicle.

The kinematics model of the bus is derived as follows [20]:

(1) $$\left\{ \begin{array}{l} \dot{x}(t)=v(t)\cos(\phi (t)+\beta (t))\\ \dot{y}(t)=v(t)\sin(\phi (t)+\beta (t))\\ \dot{\phi }(t)=v(t)\cos\beta (t)\tan\delta (t)/L\\ \dot{v}(t)=a(t) \end{array}\right.$$

where a(t) is the desired acceleration, $\beta (t)=a\tan(v_{y}/v_{x})$. The model uses δ(t), a(t) as the bus lateral and longitudinal control input, respectively.

**Figure 2:**
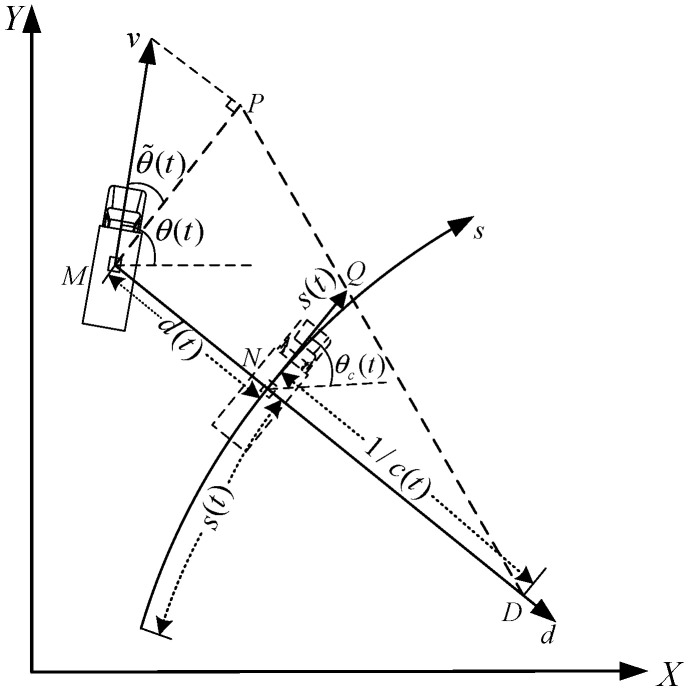
Coordinate system conversion diagram.

Model (1) considers the movement characteristics of the bus, but ignores the relationship between the desired path and the movement of the bus. In order to express the relationship between the vehicle and the path, this paper takes the desired path as a reference, and projects the model to the Frenet coordinate system. The Frenet coordinate system describes the position of the vehicle relative to the road. In the Frenet coordinate system, it is guaranteed that at each point on the road, the horizontal and vertical axes are vertical. The ordinate represents the distance traveled by the vehicle on the road, and the abscissa represents the distance vehicle deviates from the centerline. As shown in Figure 2, take the current location of the vehicle as the origin, the direction along the desired path as the s−axis, and the direction perpendicular to the desired path and pointing to the concave side of the desired path as the d−axis, to establish a Frenet coordinate system.

In Figure 2, considering the heading error of the vehicle, the longitudinal velocity of the vehicle in the Cartesian coordinate system is projected to the tangent direction along the road, and the lateral position error of the vehicle is further considered. The vehicle position and the projected vehicle speed are projected on the s−axis, and the position s(t) on the corresponding s−axis of the vehicle is regarded as the longitudinal displacement of the vehicle, and the position d(t) on the corresponding d−axis of the vehicle is regarded as the lateral displacement of the vehicle. The vector after the longitudinal velocity projection is denoted as $\dot{s}(t)$, which represents the rate of change of the longitudinal displacement of the vehicle [21].

In Figure 2, N is the closest point of the vehicle from the desired path, $\tilde{\theta }(t)$ indicates the angle at which the vehicle deviates from the expected path, $\tilde{\theta }(t)=\theta (t)-\theta_{c}(t)$, $\theta_{c}(t)$ is the heading angle at the closest point N, and c(t) is the curvature corresponding to closest point N. According to the similarity of triangle DMP and triangle DNQ (DMP and DNQ are the triangles in Figure 2), we can produce:

(2) $$\frac{1/c(t)}{1/c(t)-d(t)}=\frac{\dot{s}(t)}{v(t)\cos\tilde{\theta }(t)}$$

To further deduce the lateral and longitudinal displacement change rate of the vehicle in the Frenet coordinate system, the following expression is proposed:

(3) $$\left\{ \begin{array}{l} \dot{s}(t)=v(t)\chi (t)\\ \dot{d}(t)=v(t)\sin\tilde{\theta }(t) \end{array}\right.$$

where $\chi (t)=\cos\tilde{\theta }(t)/(1-d(t)c(t))$.

Combining the lateral and longitudinal kinematics model of the bus and the lateral and longitudinal displacement change rate of the vehicle, a combined lateral and longitudinal bus model in the Frenet coordinate system as shown in (8) is further proposed.

(4) $$\{\begin{array}{c} \dot{s}(t)=v(t)\chi (t)\\ \dot{d}(t)=v(t)\sin\tilde{\theta }(t)\\ \dot{\tilde{\theta }}(t)=v\cos\beta (t)u_{d}(t)/L-{\dot{\theta }}_{c}(t)\\ \dot{v}(t)=u_{s}(t) \end{array}$$

where ${\dot{\theta }}_{c}(t)=c(t)\dot{s}(t)$, $u_{d}(t)=\tan\delta (t)$ is the lateral control input, $u_{s}(t)=a(t)$ is the longitudinal control input.

### 2.2. Construction of Bus Platoon Error Model in Frenet Coordinate System

**Figure 3:**
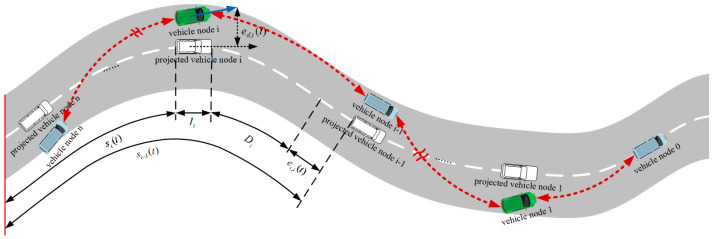
Coordinate system conversion diagram.

The description of the lateral and longitudinal errors of the bus platoon in the Frenet coordinate system is shown in Figure 3. In Figure 3, the white dashed line is the expected path, the colored vehicles are the actual vehicles in the platoon, and the white vehicles are the projections of the actual vehicles on the desired path. Among them, the longitudinal error $e_{s,i}$ of the platoon along the desired path and the lateral error $e_{d,i}$ of the platoon close to the desired path are defined, $s_{i-1}(t)$ and $s_{i}(t)$ are the longitudinal displacements of the i−1th and ith vehicles, respectively, $l_{i}$ is the length of the ith vehicle, $D_{i}(t)$ is the desired distance between vehicles, i represents the ith node, s represents the longitudinal orientation of the vehicle, and d represents the lateral orientation of the vehicle.

According to the actual longitudinal displacement between the preceding vehicle and the target vehicle and the desired distance between the vehicles, the longitudinal error is as follows:

(5) $$e_{s,i}(t)=s_{i-1}(t)-s_{i}(t)-D_{i}(t)-l_{i}$$

where $D_{i}(t)$ is the desired vehicle spacing, $s_{i-1}(t)$ and $s_{i}(t)$ are the i−1th and ith vehicle’s longitudinal displacement, $l_{i}$ is the ith vehicle’s length.

If motion planning is not considered, the constant time headway (CTH) strategy [22] is generally used to calculate the desired distance between vehicles, so that there are:

(6) $$D_{i}(t)=C+hv_{i}(t)$$

where C is the safe distance, h is the headway, $v_{i}(t)$ is obtained by the combined inertial navigation measurement or vehicle volume wheel speedometer feedback, h and C are constants.

During the manual driving of the vehicle, the driver will use a certain point in front of the vehicle as a reference to control the vehicle to travel along the desired path. Intelligent driving vehicles refer to this process and introduce the driver’s single-point preview model [23]. Ignoring the influence of the desired path curvature on the lateral preview error in a short time, the lateral error model of the bus platoon is designed according to the single-point preview model as follows:

(7) $$e_{d,i}(t)=d_{i}(t)-l_{pre}\cdot \sin({\tilde{\theta }}_{i}(t))$$

where $d_{i}(t)$ is the ith vehicle’s lateral displacement, $l_{pre}$ is the preview distance, ${\tilde{\theta }}_{i}(t)$ is the angle at which the ith vehicle deviates from the desired path.

Taking the platoon error model shown in (5) and (7) as a reference, according to the first-order hyperlocal model [24], taking the platoon lateral and longitudinal errors as the state, the nonlinear state equation of the system is constructed as follows:

(8) $$\left\{ \begin{array}{l} {\dot{e}}_{d,i}(t)=F_{d,i}(t)+b_{d,i}(t)u_{d,i}+\xi_{d,i}(t)\\ {\dot{e}}_{s,i}(t)=F_{s,i}(t)+b_{s,i}(t)u_{s,i}+\xi_{s,i}(t) \end{array}\right.$$

where $\xi_{d,i}(t)$ and $\xi_{s,i}(t)$ are the lateral and longitudinal unknown disturbance.

According to the joint kinematics model and platoon error model of the bus, the specific expressions of $F_{d,i}(t)$, $F_{s,i}(t)$, $b_{d,i}(t)$, $b_{s,i}(t)$ are as follows:

(9) $$\left\{ \begin{array}{l} F_{d,i}(t)=v_{i}(t)\sin{\tilde{\theta }}_{i}(t)-{\dot{l}}_{pre}\sin{\tilde{\theta }}_{i}(t)+l_{pre}{\dot{\theta }}_{c,i}(t)\cos{\tilde{\theta }}_{i}(t)\\ b_{d,i}(t)=-l_{pre}\cos{\tilde{\theta }}_{i}(t)v_{i}(t)/L_{i}\\ F_{s,i}(t)=v_{i-1}(t)\chi_{i-1}(t)-v_{i}(t)\chi_{i}(t)-{\dot{D}}_{i}\\ b_{s,i}(t)=-h \end{array}\right.$$

where $l_{pre}$ is the preview distance.

## 3. Bus Platoon Finite-Time Sliding Mode Control Method

### 3.1. Control Objective

Considering the rapid convergence of lateral and longitudinal errors and the stability of the platoon, this paper combines the finite-time tracking control and the cooperative control of the platoon, and express the control objectives as follows:

(1) The lateral and longitudinal tracking error of the bus platoon converges from an arbitrary state to a steady state in a finite time, and it is described as follows:

(10) $$\left\{ \begin{array}{l} \lim_{t\to T_{s,i}}\left|e_{s,i}(t)\right|=0\\ \lim_{t\to T_{d,i}}\left|e_{d,i}(t)\right|=0 \end{array}\right.$$

where $T_{s,i}$ and $T_{d,i}$ indicate the convergence time of the longitudinal error and the lateral error.

(2) When the system is subjected to unknown disturbances, the longitudinal errors of the platoon are not amplified and propagated to upstream vehicles. The description is as follows [25]:

(11) $$\left|G_{s,i}(s)\right|=\left|E_{s,i+1}(s)/E_{s,i}(s)\right|\leq 1$$

The $G_{s,i}(s)$ is the transfer function of bus platoon longitudinal error, $E_{s,i+1}(s)$, $E_{s,i}(s)$ stand for the Laplace transform of lateral and longitudinal error $e_{s,i+1}(t)$ and $e_{s,i}(t)$.

### 3.2. Design of Distributed Lateral and Longitudinal Tracking Controller Based on Finite-Time Sliding Mode

Regarding the first-order nonlinear system shown in (8), the work in [19] considered the fast convergence of the sliding mode, and put forward the fast integral terminal sliding mode surface (FITSM) as follows:

(12) $$\sigma =e{\left(t\right)}^{p_{1}/p_{2}}+\alpha_{1}\int_{0}^{t}e(\tau )d\tau +\alpha_{2}{\left(\int_{0}^{t}e(\tau )d\tau \right)}^{g_{1}/g_{2}}$$

where σ is the sliding mode variable, e(t) is the system error, $p_{1},p_{2},g_{1},g_{2}\in R^{+}$ are odd integers, and $0<p_{1}/p_{2}<1$, $g_{1}/g_{2}>p_{1}/p_{2}$, $\alpha_{1},\alpha_{2}>0$.

However, there is a negative exponential term in the control input after the derivation of FITSM, and there is a control singularity when the system error is zero.

Therefore, in order to realize the convergence of the system variables to the equilibrium position in a finite time and to avoid the control singularity problem, a non-singular integral terminal sliding mode surface (NITSM) is proposed as follows:

(13) $$\sigma =e(t)+\alpha_{1}\int_{0}^{t}e{\left(\tau \right)}^{q_{1}/q_{2}}d\tau +\alpha_{2}(\int_{0}^{t}e{\left(\tau \right)}^{q_{1}/q_{2}}d\tau {)}^{g_{1}/g_{2}}$$

where $q_{1},q_{2},g_{1},g_{2}\in R^{+}$ are odd integers, and $1<q_{1}/q_{2}<2$, $g_{1}/g_{2}>q_{2}/q_{1}$.

The NITSM converts the power term of the error into the power integral term, and solves the control singularity problem when the error is zero. NITSM can reach the same convergence rate as FITSM.

**Theorem** **1.**  *After applying NITSM to the first-order nonlinear system, if the system state trajectory achieves the sliding mode surface, the error can converge to zero in a limited time.*

**Proof.**  Because the trajectory state of the system reaches the sliding mode surface, let σ=0. According to (12), we can produce:

(14) $$e(t)+\alpha_{1}\int_{0}^{t}e{\left(\tau \right)}^{q_{1}/q_{2}}d\tau +\alpha_{2}(\int_{0}^{t}e{\left(\tau \right)}^{q_{1}/q_{2}}d\tau {)}^{g_{1}/g_{2}}=0$$

Assuming that $e_{I}(t)=\int_{0}^{t}e{\left(\tau \right)}^{q_{1}/q_{2}}d\tau$, (18) can be rewritten as (18).

(15) $${\dot{e}}_{I}{\left(t\right)}^{q_{2}/q_{1}}+\alpha_{1}e_{I}(t)+\alpha_{2}e_{I}{\left(t\right)}^{g_{1}/g_{2}}=0$$

Assuming that the time for the trajectory state to reach the sliding mode surface is $t_{r}$, according to (15), the time for $e_{I}(t)$ converge to zero is:

(16) $$t_{s}=\int_{0}^{\left|e_{I}(t_{r})\right|}\frac{1}{{\left(\alpha_{1}e_{I}(t)+\alpha_{2}e_{I}{\left(t\right)}^{g_{1}/g_{2}}\right)}^{q_{1}/q_{2}}}de_{I}$$

According to (14), when $e_{I}(t)$ converges to zero, e(t) converges to zero at the same time.

In the same way, the convergence time $t_{sf}$ of FITSM is calculated as follows:

(17) $$t_{sf}=\int_{0}^{\left|e_{I}(t_{rf})\right|}\frac{1}{{\left(\alpha_{1}e_{I}(t)+\alpha_{2}e_{I}{\left(t\right)}^{g_{1}/g_{2}}\right)}^{p_{2}/p_{1}}}de_{I}$$

If $q_{1}/q_{2}=p_{2}/p_{1}$, NITSM has the same convergence speed as FITSM. □

**Remark** **1.**  *Compared with the ITSM, NITSM can make the system state on the sliding mode surface at the initial moment by setting the initial value of the integral, thereby achieving global sliding robustness. In addition, according to (16), the larger the value of $\alpha_{1}$, $\alpha_{2}$, the faster the error convergence speed. However, as $\alpha_{1}$, $\alpha_{2}$ increase, there may be a large overshoot after the system reaches the equilibrium state. In practical applications, the selection of sliding surface parameters needs to be tuned by trial and error to balance the convergence speed of the error. In the same way, the parameters $q_{1}$, $q_{2}$, $g_{1}$, $g_{2}$ also need to be adjusted according to the error convergence speed and overshoot.*

In theory, NITSM can eliminate the approaching mode of the system. However, due to measurement errors and disturbances in the system state, it is hard to maintain the sliding mode surface in practical applications. Therefore, the work in [15] considered the finite-time reachability of the system approaching mode, and proposed a double-power approaching law with the following form:

(18) $$\dot{\sigma }=-k_{1}{\left|\sigma \right|}^{\beta_{1}}\mathrm{sgn}(\sigma )-k_{2}{\left|\sigma \right|}^{\beta_{2}}\mathrm{sgn}(\sigma )$$

where $0<\beta_{1}<1$, $\beta_{2}>1$, $k_{1},k_{2}>0$.

However, the double-power reaching law can only guarantee the convergence to the steady-state error bound in a finite time, when the system has bounded disturbances [15].

In order to eliminate the problem of finite-time convergence under bounded disturbances, this paper introduces a power integral term into the bi-power reaching law to decrease the steady-state error of the system, and proposes an adaptive power integral reaching law (APIRL), as follows:

(19) $$\left\{ \begin{array}{l} \dot{\sigma }=-k_{1}({\left|\sigma \right|}^{\beta_{1}}+{\left|\sigma \right|}^{\beta_{2}})\mathrm{sgn}(\sigma )+y\\ \dot{y}=-k_{2}(\beta_{1}{\left|\sigma \right|}^{2\beta_{1}-1}+(\beta_{1}+\beta_{2}){\left|\sigma \right|}^{\beta_{1}+\beta_{2}-1}+\beta_{2}{\left|\sigma \right|}^{2\beta_{2}-1})\mathrm{sgn}(\sigma ) \end{array}\right.$$

where $0<\beta_{1}\leq 1/2$, $\beta_{2}>1$, $k_{1}$, $k_{2}$ satisfied the following adaptive law:

(20) $$\left\{ \begin{array}{l} \sigma \neq 0,\left\{ \begin{array}{l} {\dot{k}}_{1}=\frac{\left(4n_{2}+n_{1}^{2}/2){\left({\left|\sigma \right|}^{\beta_{1}}+{\left|\sigma \right|}^{\beta_{2}}\right)}^{2}+(1+n_{1}/2\right)}{\left(1+n_{1}/2\right)}\\ k_{2}=n_{1}k_{1}/2 \end{array}\right.\\ \sigma =0,{\dot{k}}_{1}={\dot{k}}_{2}=0 \end{array}\right.$$

where $n_{1},n_{2}$ are constants.

**Lemma** **1.**  *If 0<b<1 exists, the following inequality holds:*

(21) $${\left(\left|c_{1}\right|+\left|c_{2}\right|+\cdots +\left|c_{m}\right|\right)}^{b}\leq {\left|c_{1}\right|}^{b}+{\left|c_{2}\right|}^{b}+\cdots +{\left|c_{m}\right|}^{b}$$

*where $c_{1},c_{2},\cdots ,c_{m}$ is all any real number.*

**Theorem** **2.**  *For the APIRL, when there is an unknown bounded disturbance ξ, if $k_{1}$ and $k_{2}$ fulfill the adaptive law, the sliding mode variable σ can converge to zero in finite time.*

**Proof.**  The proof is given in Appendix A. □

In order to solve the problem of the rapid convergence of the lateral and longitudinal errors of the bus platoon, this paper designs a lateral distribution and longitudinal finite-time sliding mode tracking controller based on NITSM-APIRL.

To ensure the bus platoon is stable, the design longitudinal coupling error is as follows:

(22) $${\bar{e}}_{s,i}(t)=qe_{s,i}(t)-e_{s,i+1}(t)$$

In the above formula, ${\bar{e}}_{s,i}(t)$ is the longitudinal coupling error, q>0 is the coupling factor.

Then, according to (13) and (22), the lateral and longitudinal NITSM is designed as follows:

(23) $$\left\{ \begin{array}{l} \sigma_{d,i}=e_{d,i}(t)+\alpha_{1,d,i}e_{d,i}^{I}(t)+\alpha_{2,d,i}{\left[e_{d,i}^{I}(t)\right]}^{g_{d,i}^{1}/g_{d,i}^{2}}\\ \sigma_{s,i}={\bar{e}}_{s,i}(t)+\alpha_{1,s,i}{\bar{e}}_{s,i}^{I}(t)+\alpha_{1,d,i}{\left[{\bar{e}}_{s,i}^{I}(t)\right]}^{g_{s,i}^{1}/g_{s,i}^{2}} \end{array}\right.$$

where $e_{d,i}^{I}(t)=\int_{0}^{t}e_{d,i}{\left(\tau \right)}^{q_{d,i}^{1}/q_{d,i}^{2}}d\tau$, ${\bar{e}}_{s,i}^{I}(t)=\int_{0}^{t}{\bar{e}}_{s,i}{\left(\tau \right)}^{q_{s,i}^{1}/q_{s,i}^{2}}d\tau$.

Finally, with further derivation of the sliding mode surface NITSM and combined with APIRL, the lateral and longitudinal finite-time sliding mode tracking control law is designed as follows:

(24) $$\left\{ \begin{array}{l} u_{d,i}=b_{d,i}{\left(t\right)}^{-1}\{\varphi (\sigma_{d,i})-[\alpha_{1,d,i}e_{d,i}{\left(t\right)}^{q_{d,i}^{1}/q_{d,i}^{2}}+\\ \;\;\alpha_{2,d,i}g_{d,i}^{1}{\left(e_{d,i}^{I}(t)\right)}^{g_{d,i}^{1}/g_{d,i}^{2}-1}e_{d,i}{\left(t\right)}^{q_{d,i}^{1}/q_{d,i}^{2}}/g_{d,i}^{2}+F_{d,i}(t)]\}\\ u_{s,i}=\{\varphi (\sigma_{s,i})-[\alpha_{1,s,i}{\bar{e}}_{s,i}{\left(t\right)}^{q_{s,i}^{1}/q_{s,i}^{2}}g_{s,i}^{2}+\\ \;\;\alpha_{1,s,i}g_{s,i}^{1}{\left({\bar{e}}_{s,i}^{I}(t)\right)}^{g_{s,i}^{1}/g_{s,i}^{2}}{\bar{e}}_{s,i}{\left(t\right)}^{q_{s,i}^{1}/q_{s,i}^{2}}/g_{s,i}^{2}]+\\ \;\;{\dot{e}}_{s,i+1}(t)\}/(qb_{s,i}(t))-F_{s,i}(t)/b_{s,i}(t) \end{array}\right.$$

where ${\dot{e}}_{s,i+1}(t)=F_{s,i+1}(t)+b_{s,i+1}(t)u_{s,i+1}$, $\varphi (\sigma_{d,i})$ and $\varphi (\sigma_{s,i})$ is the lateral and longitudinal APIRL, $F_{s,n+1}(t),b_{s,n+1}(t),u_{s,n+1}=0$.

**Remark** **3.**  *The distributed lateral and longitudinal finite-time sliding mode tracking controller for the bus platoon is composed of the lateral and longitudinal sliding mode surface and the finite-time sliding mode tracking control law. When the system state is on the sliding mode surface, it is in the sliding mode at this time. The system error can quickly converge to the equilibrium state, relying on the fast convergence characteristics of NITSM. When the system state leaves the sliding mode surface, it is in a trending state, and the sliding mode tracking control law forces the system to return to the sliding mode in a finite time.*

**Inference** **1.**  *The lateral and longitudinal controller design based on NITSM-APIRL can ensure that the lateral and longitudinal errors of the platoon tend to zero within a finite time and satisfy the finite-time lateral and longitudinal stability.*

**Proof.**  According to Theorem 2, the application of APIRL can ensure that the lateral and longitudinal sliding mode variable $\sigma_{s,i}$, $\sigma_{d,i}$ convergence to zero in a finite time, and at the same time the system state tends to the sliding mode surface. □

According to Theorem 1, it can be obtained that, when using NITSM and the system state is on the sliding mode surface, $\sigma_{s,i}=\sigma_{d,i}=0$, and it can be ensured that the lateral error $e_{d,i}$ and the longitudinal coupling error ${\bar{e}}_{s,i}$ convergence to zero in a finite time.

When ${\bar{e}}_{s}(t)={\left[{\bar{e}}_{s,1},{\bar{e}}_{s,2},\cdots ,{\bar{e}}_{s,n}\right]}^{T}$, $e_{s}(t)={\left[e_{s,1},e_{s,2},\cdots ,e_{s,n}\right]}^{T}$, the matrix Q is defined as:

(25) $$Q=\left[\begin{array}{ccccc} q & -1 & \cdots & 0 & 0\\ 0 & q & -1 & \cdots & 0\\ & & \ddots & & \\ 0 & 0 & \cdots & q & \\ 0 & 0 & \cdots & 0 & q \end{array}\right]$$

The coupled error can be presented as the following form:

(26) $${\bar{e}}_{s}(t)=Q\cdot e_{s}(t)$$

where q>0 is constant, matrix Q is reversible.

**Theorem** **3.**  *Assuming that 0<q<1, the system reaches the stability of the platoon, and the longitudinal error of the platoon is not amplified and propagated to the upstream vehicle.*

**Proof.**  The longitudinal error of the bus platoon coupling will tend to zero in a finite time:

(27) $${\bar{e}}_{s,i}(t)=qe_{s,i}(t)-e_{s,i+1}(t)=0$$

Pulling transformation to (44), we produce:

(28) $$qE_{s,i}(t)-E_{s,i+1}(t)=0$$

Then $E_{s,i+1}(s)/E_{s,i}(s)=q$, when 0<q<1, (11) established, and Theorem 3 is proved. □

## 4. Controller Simulation Experiment and Actual Vehicle Verification

For the purpose of verifying the effectiveness of the proposed control method, a bus platoon simulation experiment is designed based on the Trucksim/Simulink co-simulation platform, and we conduct a real vehicle verification in the Changsha National Intelligent Networked Vehicle test area.

### 4.1. Analysis of Bus Platoon Distributed Lateral and Longitudinal Tracking Control Framework

**Figure 4:**
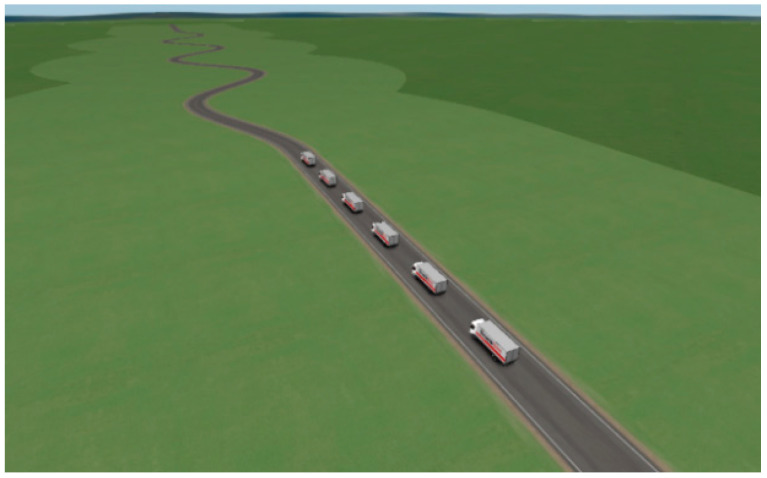
Coordinate system conversion diagram.

The lateral and longitudinal co-simulation experiment platform of the bus platoon uses Trucksim to establish a simulation scene. The vehicle dynamics parameters are shown in Table 1, and the simulation scene is shown in Figure 4.

The simulation scene is the flat, snake-shaped, pole-crossing condition (as shown in Figure 4), and the designed bus platoon is composed of a pilot bus and five following buses.

In order to verify the distributed lateral and longitudinal tracking control framework of the bus platoon proposed in this paper, we design two scenarios where the tracking error of the pilot bus/front bus is too large. Then we analyze the lateral and longitudinal error changes of the following buses, considering the chain reaction of tracking errors and the effects of lateral and longitudinal coupling.

Scenario 1: The initial lateral error of the pilot vehicle is 1.5 m, and the maximum error during driving exceeds 1 m, and the following vehicles have no initial error.

Scenario 2: The initial lateral error of the No.3 following vehicle is 1.5 m, the maximum error during driving exceeds 1 m, and the other vehicles have no initial error.

Set the initial position of the pilot bus as $s_{0}(0)=55\;m$, the initial position of the following buses as $s_{i}(0)=\left[46,37,28,19,0\right]\;m$, and the initial velocity of both the buses are 5 m/s, and the pilot velocity of the bus changes as follows:

(29) $$v_{0}(t)=\left\{ \begin{array}{ll} 5\;m/s, & \;0\leq t\leq 10\\ (t-10)+5\;m/s, & \;10<t\leq 25\\ 20\;m/s, & \;25<t\leq 45\\ -0.5(t-45)+20\;m/s, & 45<t\leq 60\\ 12.5\;m/s, & t>60 \end{array}\right.$$

The simulation time is 70 s, the step length is 0.01 s, and the model and controller parameters are shown in Table 2.

**Figure 5:**
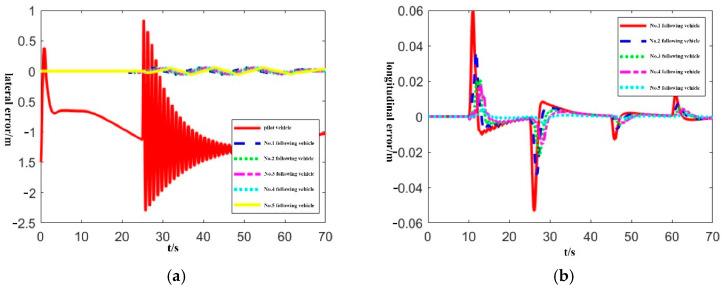
Lateral and longitudinal error curve in scenario 1. (**a**) Platoon lateral error; (**b**) Platoon longitudinal error.

**Figure 6:**
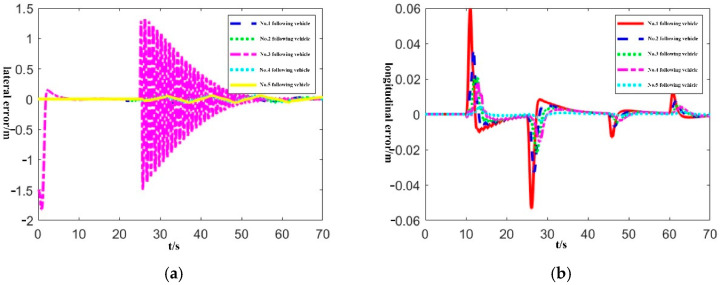
Lateral and longitudinal error curve in scenario 2. (**a**) Platoon lateral error; (**b**) Platoon longitudinal error.

In the simulation, because the bus platoon is homogeneous, all controllers use the same parameters. The experimental results are shown in Figure 5 and Figure 6. In Figure 5, the pilot vehicle has a large tracking error, and the lateral error fails to converge to zero. However, according to Figure 5a, it can be seen that the following vehicles do not have a “chain reaction” and still maintain a small tracking error. According to Figure 5b, the lateral and longitudinal control framework proposed in this paper is adopted, and the lateral loss control of the pilot vehicle does not affect the control of the longitudinal distance between vehicles. In Figure 6, the tracking error of the No.3 following vehicle is large, and the lateral error oscillates violently. According to Figure 6a, the lateral error of the other vehicles is almost unaffected, and the higher control accuracy can still be maintained. From Figure 6b, it can be found that the following vehicle with losing lateral control does not affect the longitudinal control of the platoon.

### 4.2. Comparison and Analysis of Controller Performance under Different Control Methods

To verify the performance of the controller, a joint lateral and longitudinal simulation experiment of the bus platoon in the curve scene is designed, and then three different control methods are compared and analyzed.

Control method 1: The finite-time sliding mode tracking controller based on NITSM-APIRL, which was designed in this paper.

Control method 2: The high-order sliding mode tracking controller with improved super spiral algorithm, which was designed by [26].

Control method 3: The distributed adaptive integral sliding mode tracking controller, which was designed by [27].

**Figure 7:**
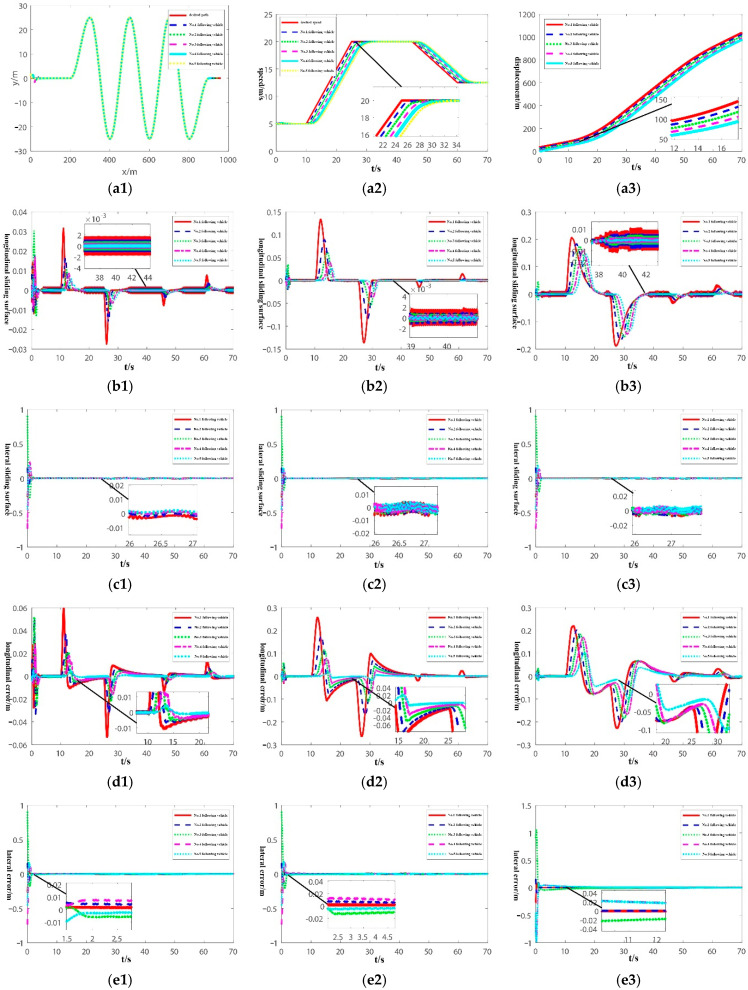

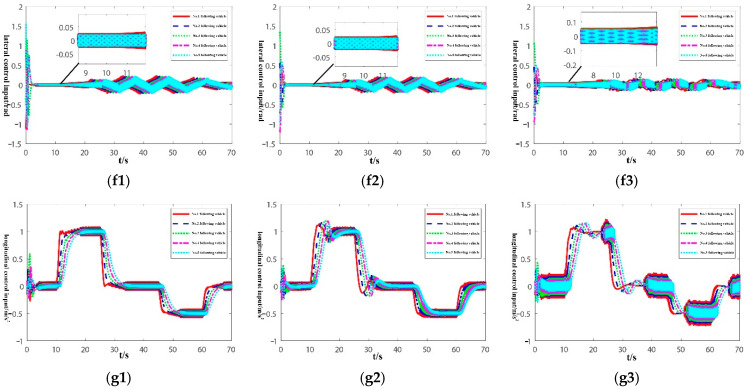
Experimental results of lateral and longitudinal control of bus platoon under different control methods.

The initial longitudinal displacement and initial speed of the pilot vehicle/following vehicles are the same as those in Section 4.1. Assuming that the initial heading error of the following vehicles is ${\tilde{\theta }}_{i}(0)=\left[0,0.3,-0.25,0.25,-0.2\right]\;\mathrm{rad}$, the initial lateral displacement of the following vehicles is $d_{i}(0)=[0.55,-0.9,-1.25,0.95,1.25]\;m$, and the lateral and longitudinal disturbance $0.03\sin(2\pi t)e^{(-t+10)/5},t\geq 10$ are applied. The experimental results are shown in Figure 7.

Figure 7(a1–a3) are the lateral and longitudinal motion curves of the bus platoon when the control method 1 is applied. It is clear that the expected path is a serpentine through-rod condition, and the initial lateral error can gradually converge until a steady state is reached in a short time. The expected velocity includes three state of motion: constant speed, acceleration, and deceleration. Moreover, the distance between vehicles is proportional to the velocity of the vehicles.

Figure 7(b1–b3) show the longitudinal sliding surface of the bus platoon when three control methods are applied. Figure 7(c1–c3) show the lateral sliding surface of the bus platoon when three control methods are applied. Due to the existence of the initial lateral error, the lateral sliding surface has the initial error, and the initial error can converge to a steady state in a short period. Compared with three control methods, it can be seen that the APIRL can ensure the rapid convergence of the system in the approaching mode, and the control method 1 has the fastest convergence speed in the approaching mode, and the second is the method 2. The method 3 has the slowest convergence speed. In addition, both methods 1 and 2 consider the influence of any unknown disturbances on the steady-state error of the system. According to the enlarged Figure 7(b1–b3), it can be highlighted that the steady-state errors of methods 1 and 2 are all close to zero, and the steady-state error of method 3 is relatively large, and it fails to completely converge to the steady state. Moreover, according to Figure 7(b3,c3), the state trajectory reaches the sliding surface, then traverses back and forth on both sides of the sliding surface by applying the method 3, which is similar to the simulation results in [28]. In the application of methods 1 and 2, the sliding mode chattering is suppressed because the sliding mode switching item is not contained in the control input, and the sliding mode switching item is contained in the method 3, so the chattering is obvious.

Figure 7(d1–d3) show the longitudinal error of the bus platoon when three control methods are applied. The longitudinal error after the deviation has the fastest convergence speed by applying control method 1, the second is method 2, and method 3 has the slowest convergence speed. In addition, the controller designed in this paper introduces a power integral term and an integral terminal sliding surface, so the steady-state error can be reduced. The maximum longitudinal error is about 0.06 m by applying the control method 1, the maximum longitudinal error is about 0.25 m by applying method 2, and the maximum longitudinal error is about 0.23 m by applying method 3. Moreover, the control method 1 contains the double-power approaching term, so it has a faster convergence rate than the single-power approaching term in the method 2, and the method 3 cannot guarantee the finite-time convergence of the sliding mode, so the control method 1 has the fastest longitudinal convergence speed. In addition, according to the experimental results, when the upstream vehicle has a longitudinal error, the error will gradually decrease in the process of propagation to the downstream vehicle. The coupled platoon longitudinal error can ensure the stability of the platoon.

Figure 7(e1–e3) show the lateral error of the bus platoon when three control methods are applied. It can be concluded from the experimental results that the initial lateral error converges to a steady state at about 1.5 s by applying method 1, the initial lateral error converges to a steady state at about 2.5 s by applying method 2, and the initial lateral error converges to a steady state at about 10 s by applying method 3. Obviously, method 1 has the fastest lateral convergence speed. Moreover, it can be seen that during the movement of the platoon, the presence of the lateral error does not affect the convergence of the longitudinal error. Figure 7(f1–f3) show the lateral control input of the bus platoon when three control methods are applied. Figure 7(g1–g3) show the longitudinal control input of the bus platoon when three control methods are applied. The control input is relatively smooth when applying the control methods 1 and 2; the lateral control input does not exceed 1.5 rad and the longitudinal control input does not exceed 1.5 ${m/s}^{2}$, thereby meeting the lateral and longitudinal actuator action restrictions.

In summary, the lateral and longitudinal control performance of the bus platoon is shown in Table 3. According to Table 3, the application of the method 1 can ensure the lateral and longitudinal finite-time stability and the platoon stability, the lateral and longitudinal errors can converge to the stable state at the fastest speed with the smallest steady-state error, and the application of the method 1 can effectively inhibit the sliding mode chattering.

### 4.3. Real Vehicle Experiment of Intelligent Bus Platoon

**Figure 8:**
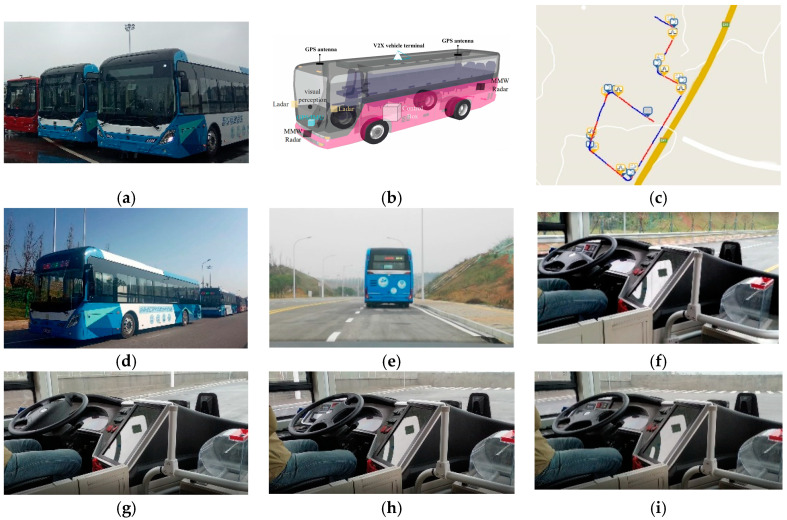
Real vehicle test platform and process.

In this paper, three intelligent buses are used as the research object, and the experiment is carried out in the National Intelligent Connected Vehicle (Changsha) test area. The experimental platform and process are shown in Figure 8.

Figure 8a is the experiment platform for the bus platoon, which is composed of three pure electric buses that have completed intelligent transformation. Figure 8b is a configuration figure of the vehicle sensors. Each vehicle in the platoon is equipped with a GPS/IMU, V2X terminal, camera, lidar, and millimeter wave radar. The GPS/IMU provides real-time pose information about the vehicle, and the V2X terminal is used for the communication between the vehicles, transmitting the model and control information about the following vehicle, and transmitting the displacement and speed of the preceding vehicle. The camera, lidar, and millimeter wave radar are mainly used for obstacle detection and recognition, providing references for vehicle decision-making and planning, and generating the desired paths. Figure 8c is a road model constructed in the Changsha test area, which includes the global desired path, intersections, exit/entry points, and other information. Figure 8d is the initial bus platoon, composed of three intelligent buses. Figure 8e is the forward vision of the following vehicle. It can be seen from the steering wheel angle that Figure 8f is a screenshot of the vehicle control process in the straight line scene, and Figure 8g–i are the screenshots of the vehicle control process in the curve scene.

**Figure 9:**
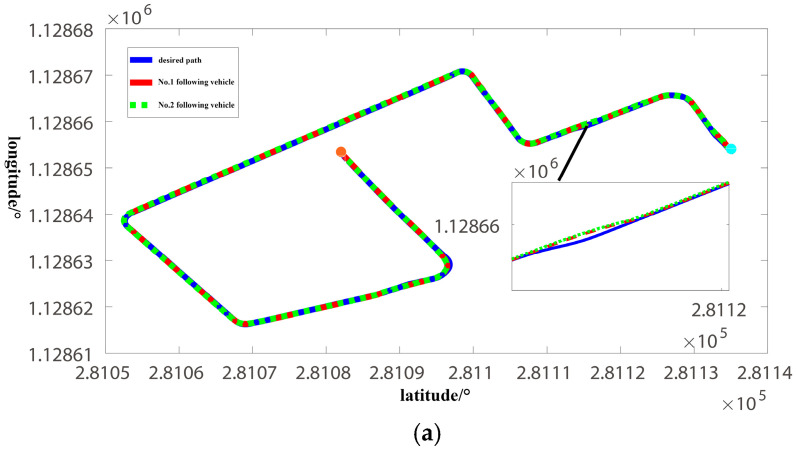

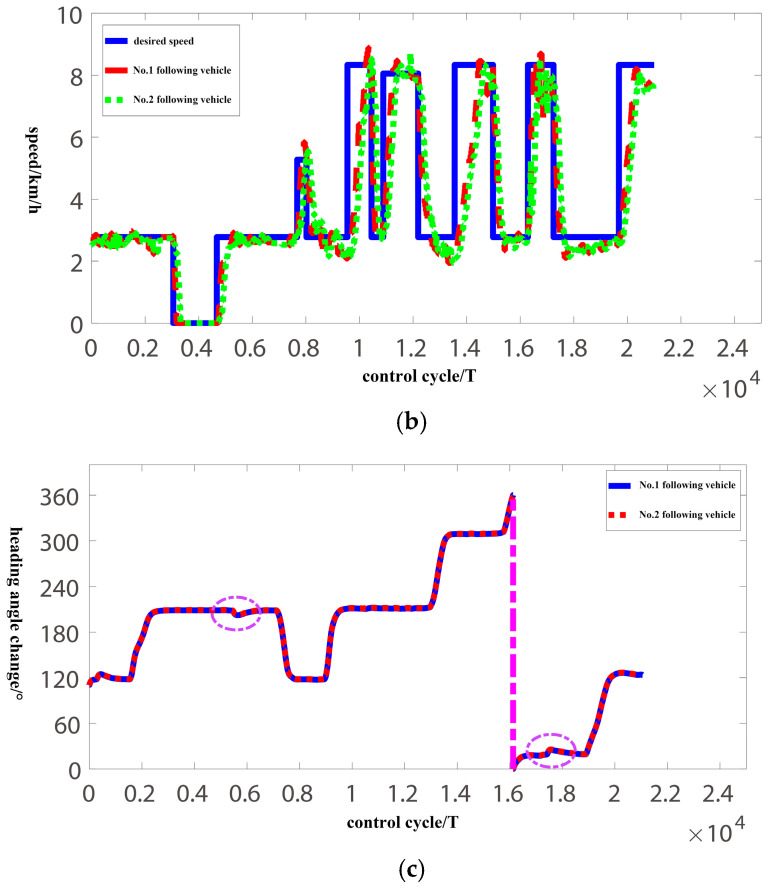
Lateral and longitudinal movement trajectory of bus platoon. (**a**) Path following curve; (**b**) Speed tracking curve; (**c**) Vehicle heading change.

**Figure 10:**
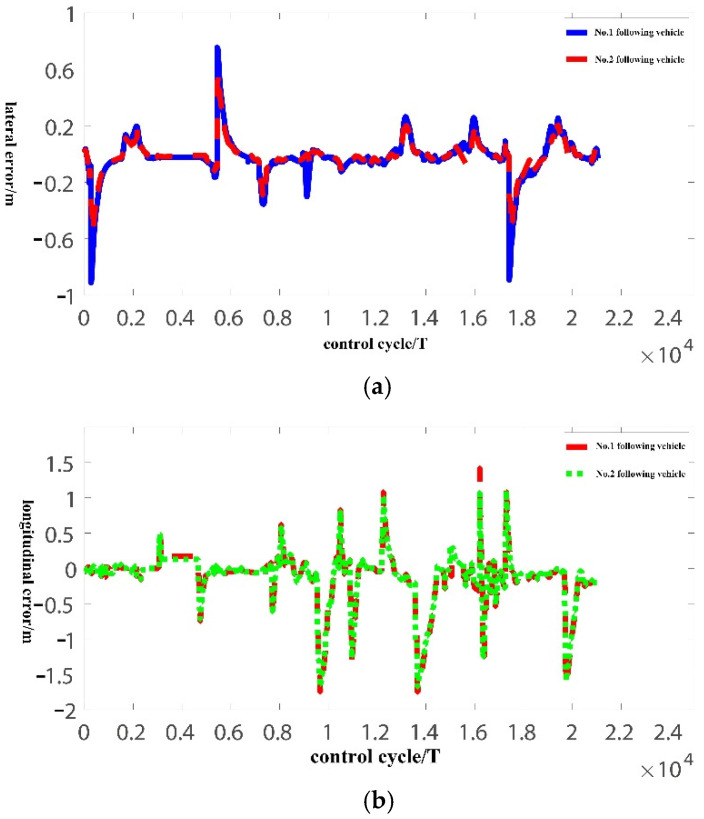
Lateral and longitudinal error curve of bus platoon. (**a**) Platoon lateral error curve; (**b**) Platoon longitudinal error curve.

**Figure 11:**
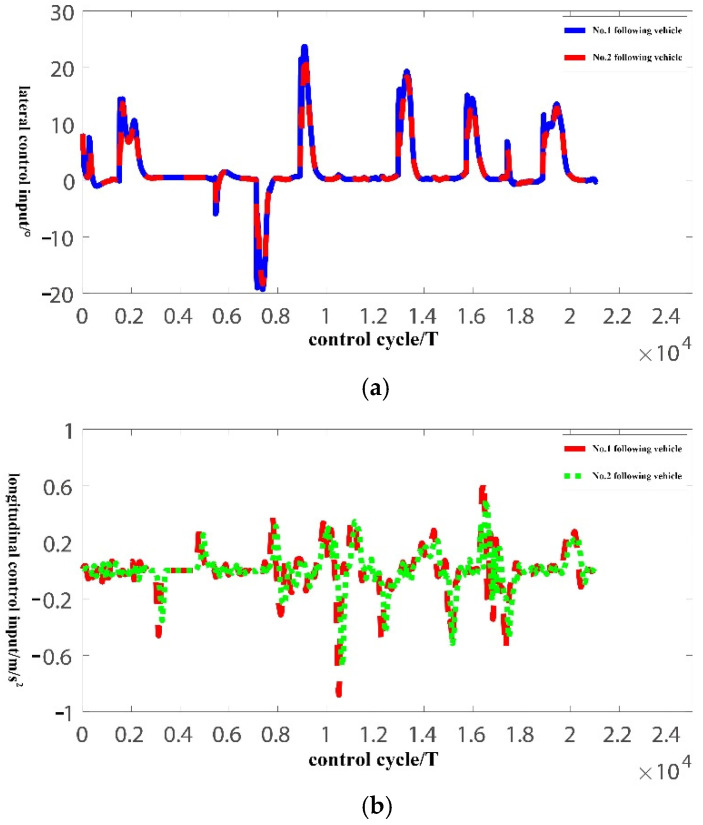
Lateral and longitudinal error curve of bus platoon. (**a**) Platoon lateral control input; (**b**) Platoon longitudinal control input.

The pilot vehicle is running in the automatic driving mode. The two following vehicles use the distributed lateral and longitudinal finite-time sliding mode controller designed in this paper. The initial vehicle speed, initial lateral position error, and longitudinal vehicle spacing error are zero, and the control cycle is 50 ms. The experiment results are shown in Figure 9, Figure 10 and Figure 11.

Figure 9a shows the driving path of the bus platoon on the test route; the total length is about 3.5 km, and it includes six right-angle bends. Figure 9b shows the speed tracking curve; the pilot vehicle detects an obstacle in front of it at 3000~5000 T, and as the desired speed is 0, the following vehicles will decelerate and stop in turn. The vehicle will start again after the obstacle disappears. In addition, when the vehicle is traveling on a straight road, the desired speed is 8.33 m/s, and when it is traveling on a curve, the desired speed is 2.78 m/s. During the experiment, there is no human intervention in the whole process, and the bus platoon designed in this paper has a high level of intelligence. Figure 9c shows the vehicle heading change curve in the northeast sky coordinate system. The vehicle heading angle varies from 0° to 360°, and the east direction is 0° or 360°. The initial vehicle heading is about 120°. The six moments of 0.18, 0.7, 0.9, 1.3, 1.58, and 1.9 (×10,000 T) in Figure 9c correspond to the six curves in Figure 9a, and the vehicle heading angle has changed significantly. In addition, at 0.55 and 1.75 (×10,000 T), the vehicle starts to change lanes and the vehicle heading angle also changes. At 1.6 (×10,000 T), the vehicle finishes the first lap and reaches the east direction (heading angle is 360°), and then the vehicle continues to move from the east direction (heading angle is 0°) for the second lap.

Figure 10a shows the platoon lateral error curve. According to Figure 10a, due to the initial heading error, the lateral position error of the platoon first increases and then decreases in the first moment; the maximum lateral error of the platoon under the curve is about 0.4 m, and the maximum error under the straight is about 0.2 m. In addition, at the two moments of 0.58 and 1.75 (×10,000 T), the lateral error is close to 1 m, because the lane-changing feature points are not marked in the feature map shown in Figure 9c, and the lateral control is still performed according to the parameters of straight-line driving, resulting in an excessive lateral error. Because the lateral steady-state error of the platoon does not exceed 0.5 m, it complies with the lateral control standard of the autonomous driving function test procedure for intelligent networked vehicles (trial). At the same time, comparing Figure 9c and Figure 10a, it can be seen that the lateral error of the platoon begins to increase when turning, and after returning to the straight road, the error converges quickly without an obvious overshoot, which meets the rapidity requirement of bus platoon control.

Figure 10b shows the platoon longitudinal error curve. Compared with Figure 9b and Figure 10b, we can see that due to the change of the desired speed, there is an excessive error in longitudinal vehicle spacing. The desired vehicle spacing obtained based on the CTH strategy changes quickly, while the actual vehicle spacing requires a period to track the desired distance between vehicles. According to Figure 10b, the longitudinal vehicle spacing error does not exceed 2 m. The longitudinal safety distance is set to 10 m during the actual bus platoon experiment, and the longitudinal error does not exceed 20% of the safety distance, so it conforms to the longitudinal control standard of the intelligent networked vehicle automatic driving function test procedure. Due to the change of desired speed, the longitudinal error increases, and the longitudinal error will fast converge to a steady state after the desired speed stabilizes, which meets the rapidity requirements of the bus platoon longitudinal control. In Figure 10b, the longitudinal error of the No.2 following vehicle is smaller than that of the No.1 following vehicle, so the longitudinal error is not amplified and propagated to the upstream vehicle, which meets the requirements of platoon stability.

Figure 11 shows the lateral and longitudinal control input of the vehicle platoon. Considering that the controller in this paper cannot completely eliminate the sliding mode chattering, so as to avoid the frequent action of the actuator, the hyperbolic tangent function is used instead of the sign function in the real vehicle experiment to further reduce the influence of sliding mode chattering. According to Figure 11a,b, the lateral and longitudinal control input are relatively smooth, the action range is small, the lateral control input does not exceed 25°, and the longitudinal control input does not exceed 1.

## 5. Conclusions

In order to reduce the chain reaction of tracking error and the effect of lateral and longitudinal coupling, this paper uses projection transformation to establish a decoupled distributed lateral and longitudinal finite-time sliding mode tracking control framework. Considering the rapid error convergence, a non-singular integral terminal sliding mode surface (NITSM) is designed to ensure the finite-time convergence of the system sliding mode, and an adaptive power integral reaching law (APIRL) is proposed to ensure the finite-time reachability of the system approaching modes. Based on NITSM-APIRL, a distributed lateral and longitudinal finite-time sliding mode tracking controller for the bus platoon is designed, and a Lyapunov function is constructed to analyze the system finite-time stability and platoon stability. Based on the Trucksim/Simulink joint simulation experiment platform, the control performance of the proposed method and the existing methods are compared. The results of the simulation indicate that the method proposed in this paper can ensure the stability of the platoon, achieve rapid error convergence, and avoid the influence of vehicle lateral movement on the longitudinal error. The actual vehicle verification was carried out in the Changsha National Intelligent Connected Vehicle testing zone, and the intelligent networked vehicle test was successfully passed, which verified the practicability of the designed controller. However, due to the platoon size is small and vehicle speed is low, the designed controller has not considered the impact of communication delay, and it will be improved in the next research.

## Figures and Tables

**Table 1:** Vehicle parameters.

Parameters	Value	Unit
$M_{i}$	4456	kg
$l_{i}$	10	m
$L_{i}$	5	m
$\rho_{i}$	23.1	-
$A_{i}$	6.8	$m^{2}$
$\varsigma_{i}$	0.96	-
$R_{i}$	0.52	m

**Table 2:** Model and controller parameters.

Parameters	Value	Parameters	Value	Parameters	Value
$l_{\min}$	2 m	$l_{\max}$	5 m	T	0.2 s
$v_{\min}$	2 m/s	$v_{\max}$	20 m/s	h	0.8 s
C	5 m	q	0.6	$\alpha_{1,d,i}$	0.1
$\alpha_{2,d,i}$	0.1	$p_{d,i}^{1}$	5	$p_{d,i}^{2}$	9
$g_{d,i}^{1}$	7	$g_{d,i}^{2}$	3	$n_{d,i}^{1}$	1.3
$n_{d,i}^{2}$	1.8	$\alpha_{1,s,i}$	0.2	$\alpha_{2,s,i}$	0.1
$p_{s,i}^{1}$	3	$p_{s,i}^{2}$	5	$g_{s,i}^{1}$	9
$g_{s,i}^{2}$	5	$n_{s,i}^{1}$	1.5	$n_{s,i}^{2}$	2.2
$\beta_{d,i}^{1}$	0.5	$\beta_{d,i}^{2}$	1.5	$\beta_{s,i}^{1}$	0.5
$\beta_{s,i}^{2}$	1.5	$k_{d,i}^{1}$	1	$k_{s,i}^{1}$	1.5

**Table 3:** Lateral and longitudinal control performance of bus platoon.

Performance	Distributed Lateral and Longitudinal Controller		
	Control Plan 1 (This Article)	Control Plan 2	Control Plan 3
limited time available	Yes	Yes	Yes
limited time to stabilize	Yes	Yes	No
stable platoon	Yes	Yes	Yes
longitudinal convergence time	10 s	15 s	>15 s
lateral convergence time	1.5 s	2.5 s	10 s
maximum longitudinal error	0.06 m	0.25 m	0.23 m
maximum lateral error	0.03 m	0.06 m	0.05 m
chattering degree	weaker	weaker	stronger
control input	smoother	smoother	not smooth

## Data Availability

Not applicable.

## Appendix A

**Proof** **of** **Theorem** **1.** When there is a differentiable bounded disturbance ξ, substitute it into APIRL and the system dynamic equation can be written as follows:

(A1) $$\left\{ \begin{array}{l} \dot{\sigma }=-k_{1}({\left|\sigma \right|}^{\beta_{1}}+{\left|\sigma \right|}^{\beta_{2}})\mathrm{sgn}(\sigma )+y\\ \dot{y}=-k_{2}(\beta_{1}{\left|\sigma \right|}^{2\beta_{1}-1}+(\beta_{1}+\beta_{2}){\left|\sigma \right|}^{\beta_{1}+\beta_{2}-1}+\beta_{2}{\left|\sigma \right|}^{2\beta_{2}-1})\mathrm{sgn}(\sigma )+\dot{\xi } \end{array}\right.$$

To prove the stability of the system, define the Lyapunov candidate function as follows:

(A2) $$V_{0}=NPN^{T},N=\left[\begin{array}{cc} \left({\left|\sigma \right|}^{\beta_{1}}+{\left|\sigma \right|}^{\beta_{2}})\mathrm{sgn}(\sigma \right) & y \end{array}\right]$$

Because the matrix P is a symmetric positive definite matrix, $V_{0}>0$ satisfies:

(A3) $$\lambda_{\min}\left\{P\right\}{\left\|N\right\|}_{2}^{2}\leq V_{0}\leq \lambda_{\max}\left\{P\right\}{\left\|N\right\|}_{2}^{2}$$

where $\lambda_{\min}\left\{\cdot \right\}$, $\lambda_{\max}\left\{\cdot \right\}$ are the minimum and maximum eigenvalues. The derivative of N is:

(A4) $$\dot{N}=-(\beta_{1}{\left|\sigma \right|}^{\beta_{1}-1}+\beta_{2}{\left|\sigma \right|}^{\beta_{2}-1})NK+\Phi \dot{\xi }$$

where $K=\left[\begin{array}{cc} k_{1} & k_{2}\\ -1 & 0 \end{array}\right]$, $\Phi =\left[\begin{array}{cc} 0 & 1 \end{array}\right]$. Then the derivative of $V_{0}$ is:

(A5) $$\begin{array}{l} {\dot{V}}_{0}=NPN^{T}=2\dot{N}PN^{T}\\ \;=-2[(\beta_{1}{\left|\sigma \right|}^{\beta_{1}-1}+\beta_{2}{\left|\sigma \right|}^{\beta_{2}-1})NK-\Phi \dot{\xi }]PN^{T}\\ \;=-\beta_{1}{\left|\sigma \right|}^{\beta_{1}-1}NRN^{T}-(2\beta_{2}{\left|\sigma \right|}^{\beta_{2}-1}NK-2\Phi \dot{\xi })PN^{T} \end{array}$$

If $(2\beta_{2}{\left|\sigma \right|}^{\beta_{2}-1}NK-2\Phi \dot{\xi })PN^{T}\geq 0$, ${\dot{V}}_{0}$ can be rewritten as:

(A6) $${\dot{V}}_{0}\leq -\beta_{1}{\left|\sigma \right|}^{\beta_{1}-1}NRN^{T}$$

Because $0<\beta_{1}\leq 1/2$, $\beta_{2}>1$, then

(A7) $${\left\|N\right\|}_{2}\geq {\left|\sigma \right|}^{1-\beta_{1}}$$

If the matrix R is a positive definite matrix, then:

(A8) $${\dot{V}}_{0}\leq -\eta V_{0}^{1/2},\eta =-\beta_{1}\lambda_{\min}\left\{R\right\}/\lambda_{\max}^{1/2}\left\{P\right\}$$

According to Lemma 1, σ can converge to zero in a finite time, and the convergence time is:

(A9) $$t_{0}\leq -2V_{0}/\eta$$

However, the first-order derivative of the disturbance $\dot{\xi }$ is difficult to be observed in real time, and the value range of the parameters $k_{1}$ and $k_{2}$ are difficult to determine, so the values of $k_{1}$ and $k_{2}$ are adjusted in real time to adapt to the influence of the unknown disturbance. For this reason, the Lyapunov candidate function is redesigned as follows:

(A10) $$V=V_{0}+{\left(k_{1}-k_{1}^{*}\right)}^{2}/2+{\left(k_{2}-k_{2}^{*}\right)}^{2}/2$$

where $k_{1}^{*}>k_{1}$, $k_{2}^{*}>k_{2}$ is constant and satisfies $(2\beta_{2}{\left|\sigma \right|}^{\beta_{2}-1}NK^{*}-2\Phi \dot{\xi })PN^{T}\geq 0$, and R is a positive definite matrix. Further derivation of V, then

(A11) $$\begin{array}{l} \dot{V}={\dot{V}}_{0}+(k_{1}-k_{1}^{*}){\dot{k}}_{1}+(k_{2}-k_{2}^{*}){\dot{k}}_{2}\\ \;=-\beta_{1}{\left|\sigma \right|}^{\beta_{1}-1}N(KP+PK^{T})N^{T}\\ \;\;-(2\beta_{2}{\left|\sigma \right|}^{\beta_{2}-1}NK-2\Phi \dot{\xi })PN^{T}\\ \;\;+(k_{1}-k_{1}^{*}){\dot{k}}_{1}+(k_{2}-k_{2}^{*}){\dot{k}}_{2}\\ \;=-\beta_{1}{\left|\sigma \right|}^{\beta_{1}-1}N((K^{*}-\tilde{K})P+P{\left(K^{*}-\tilde{K}\right)}^{T})N^{T}\\ \;\;-(2\beta_{2}{\left|\sigma \right|}^{\beta_{2}-1}N(K^{*}-\tilde{K})-2\Phi \dot{\xi })PN^{T}\\ \;\;+(k_{1}-k_{1}^{*}){\dot{k}}_{1}+(k_{2}-k_{2}^{*}){\dot{k}}_{2}\\ \;=-\beta_{1}{\left|\sigma \right|}^{\beta_{1}-1}N(K^{*}P+PK^{*T})N^{T}\\ \;\;-(2\beta_{2}{\left|\sigma \right|}^{\beta_{2}-1}NK^{*}-2\Phi \dot{\xi })PN^{T}\\ \;\;+(\beta_{1}{\left|\sigma \right|}^{\beta_{1}-1}+\beta_{2}{\left|\sigma \right|}^{\beta_{2}-1})N(\tilde{K}P+P{\tilde{K}}^{T})N^{T}\\ \;\;+(k_{1}-k_{1}^{*}){\dot{k}}_{1}+(k_{2}-k_{2}^{*}){\dot{k}}_{2} \end{array}$$

We can further obtain:

(A12) $$\begin{array}{l} V\leq -\eta V_{0}^{1/2}+(k_{1}-k_{1}^{*}){\dot{k}}_{1}+(k_{2}-k_{2}^{*}){\dot{k}}_{2}\\ \;\;\;+(\beta_{1}{\left|\sigma \right|}^{\beta_{1}-1}+\beta_{2}{\left|\sigma \right|}^{\beta_{2}-1})N(\tilde{K}P+P{\tilde{K}}^{T})N^{T} \end{array}$$

where $K^{*}=\left[\begin{array}{cc} k_{1}^{*} & k_{2}^{*}\\ -1 & 0 \end{array}\right]$, $\tilde{K}=K^{*}-K=\left[\begin{array}{cc} {\tilde{k}}_{1} & {\tilde{k}}_{2}\\ 0 & 0 \end{array}\right]$. Substitute the adaptive law, and reduce it:

(A13) $$\dot{V}\leq -\eta V_{0}^{1/2}-({\tilde{k}}_{1}+{\tilde{k}}_{2})$$

According to Lemma 2, the following is gained:

(A14) $$\dot{V}\leq -{\left(\eta^{2}V_{0}+{\tilde{k}}_{1}^{2}+{\tilde{k}}_{2}^{2}\right)}^{1/2}=-\gamma V^{1/2}$$

where $\gamma =\min(\eta ,\sqrt{2})$. Similarly, according to Lemma 1, the sliding mode variable σ can converge to zero in a finite time, and the convergence time is estimated as:

(A15) $$t_{r}\leq -2V/\gamma$$

In summary, the APIRL designed in this paper can ensure the finite-time reachability of the system under differentiable and bounded disturbances. Theorem 2 is proved. According to (A14), when $\eta >\sqrt{2}$, APIRL reaches the fastest convergence rate. When $\eta <\sqrt{2}$, it can be concluded from (A8) that the convergence velocity of the system is positively correlated with $\beta_{1}$, $\lambda_{\min}\{R\}$ and negatively correlated with $\lambda_{\max}\left\{P\right\}$. Therefore, the eigenvalues of the matrix R and the matrix P can be preset under the condition of no disturbance, and the initial value of $k_{1}$, $k_{2}$ and the parameter value of $n_{1}$, $n_{2}$ in the adaptive law can be further obtained. □
